# Sestrin2 Overexpression Inhibits Proliferation and Epithelial–Mesenchymal Transition and Induces Autophagy Through the AMPK/mTOR Signaling Pathway in Human Prostate Cancer Cells

**DOI:** 10.1155/proc/8842203

**Published:** 2025-07-07

**Authors:** Yae-Ji Kim, Hui-Ju Lee, Kyung-Hyun Kim, Geum-Lan Hong, Ju-Young Jung

**Affiliations:** Department of Veterinary Medicine & Institute of Veterinary Science, Chungnam National University, Daejeon 34134, Republic of Korea

**Keywords:** autophagy, metastasis, proliferation, prostate cancer, SESN2

## Abstract

**Background:** Prostate cancer is the most common malignancy in men. Sestrin2 (SESN2) has antitumor activity against several types of cancers. However, the effect of SESN2 on prostate cancer is not well known. In this study, we showed that SESN2 inhibits human prostate cancer.

**Materials and Methods:** To investigate the contribution of Sestrin2 to prostate cancer, we performed a bioinformatic analysis of the Cancer Genome Atlas database and Gene Expression Profiling Interactive Analysis. Using the Sestrin2 overexpression vector, we identified proliferation, migration, and invasion in prostate cancer cells. Furthermore, the effect of Sestrin2 on autophagy was confirmed by Western blot analysis and immunofluorescence staining.

**Results:** We showed that expression of SESN2 was reduced in prostate cancer tissues and cell lines, and low expression of SESN2 correlated with decreased survival in prostate cancer patients. We have shown that SESN2 inhibits cell viability and cell proliferation-related protein levels in PC3 and DU145 prostate cancer cells. SESN2 inhibited EMT-related protein and migration and invasion levels. SESN2 promoted autophagy by increasing autophagy-related protein levels and LC3-positive cells. SESN2 increased pAMPK and decreased pmTOR protein levels. Furthermore, we used rapamycin, an mTOR inhibitor, to determine whether the AMPK/mTOR signaling pathway regulates autophagy in prostate cancer cells.

**Conclusion:** Our study suggests that SESN2 inhibits prostate cancer cells by inducing autophagy through the AMPK/mTOR signaling pathway. These results indicate that SESN2 might be a novel target for prostate cancer.

## 1. Introduction

Prostate cancer is one of the leading causes of death for men in the United States and the fourth most common cancer worldwide [1]. Early prostate cancer has no specific symptoms, and in most cases, it has already progressed by the time of discovery. Recently, the incidence of advanced prostate cancer has increased. Although our understanding and treatment of prostate cancer have improved substantially over the past few years, the molecular regulation of prostate cancer cell growth remains unclear [2]. Therefore, research on the underlying mechanisms involved, including prostate cancer incidence and recurrence, is urgently required to identify new targets for prostate cancer treatment.

Autophagy is a cell regeneration system that decomposes damaged or unnecessary proteins and organelles [3]. It is a multistep, complicated process regulated by multiple signaling pathways [4]. Various human disorders, including cancer, are associated with autophagy dysregulation [5]. In addition, the research indicates that autophagy plays an essential part in tumor cell proliferation and differentiation [6]. Although many studies have investigated autophagy in prostate cancer, the precise role of autophagy in the development of prostate cancer remains unclear.

Sestrins (SESNs) belong to the stress-induced protein family found in mammals and consist of SESN1, SESN2, and SESN3 [7]. In particular, SESN2 is a conserved antioxidant and metabolic regulator downstream of p53 [8]. SESN2 may serve as potential therapeutic targets for metabolic disorders, cardiovascular and neurodegenerative diseases, and cancer [9, 10]. In breast cancer, SESN2 regulates AMPK subunit expression and modulates responses to ionizing radiation, while in colorectal cancer, it inhibits cell proliferation via AMPK/mTORC1 pathway activation [11, 12]. Additionally, SESN2 suppresses NK-92 cell-mediated cytotoxic activity through AMPK and mTORC1 signaling in ovarian cancer cells [13]. Although SESN2 exerts tumor-suppressive effects in various cancers, its role and underlying mechanisms in prostate cancer remain largely unexplored.

In this study, we identified the expression of SESN2 and the regulation of autophagy in PC3 and DU145 cells. In addition, we investigated the signaling pathway of SESN2 in prostate cancer cells.

## 2. Materials and Methods

### 2.1. Bioinformatics Analysis

Prostate cancer patient tissue data were analyzed from the Human Protein Atlas database (https://www.proteinatlas.org). SESN2 gene expression data were analyzed by Gene Expression Profiling Interactive Analysis (GEPIA; http://gepia.cancer-pku.cn). GEPIA is a web server that customizes RNA sequencing expression data from 152 normal and 492 tumor samples based on large genome and gene expression databases, including The Cancer Genome Atlas and Genotype-Tissue Expression. These databases were used to generate survival curves, including overall and disease-free survival based on SESN2 expression for prostate cancer.

### 2.2. Cell Culture and Treatment

The normal human prostate cell line, RWPE-1, and human prostate carcinoma cell lines, LNCaP and PC3, were purchased from the American Type Culture Collection (ATCC, VA, USA). The human prostate carcinoma cell line, DU145, was purchased from the Korean Cell Line Bank (Seoul National University, South Korea). RWPE-1 cells were cultured in keratinocyte serum-free medium (Gibco, NY, USA). LNCaP, PC3, and DU145 were cultured in RPMI-1640 medium (Gibco) supplemented with 1% penicillin/streptomycin (Sigma-Aldrich, MO, USA) and 10% fetal bovine serum (FBS; Gibco). All cells were incubated in a humidified atmosphere of 5% CO_2_ at 37°C. Rapamycin was purchased from Sigma-Aldrich. Rapamycin was treated at a concentration of 10 nM for 48 h.

### 2.3. Cell Transfection

To overexpress SESN2, we used an EGFP control vector containing the SESN2 gene overexpression vector (VB900019-2155kdn). The control group used the EGFP control vector. SESN2 overexpression vector and EGFP control vector were purchased from Vector Builder (Illinois, USA). PC3 and DU145 cells were seeded in 6-well plates at a density of 5 × 105 cells/mL for 24 h, followed by transfection of the SESN2 vector and control vector. We transfected them with a transfection reagent, Lipofectamine 2000 (Thermo Fisher Scientific, MA, USA), according to the manufacturer's protocol.

### 2.4. Western Blot Analysis

All cells were washed three times with cold phosphate-buffered saline (PBS) and lysed in RIPA lysis buffer (Cell Signaling Technology, MA, USA) on ice. Cell lysates were centrifuged at 12,000 rpm for 15 min at 4°C. Extracted proteins were quantified using a BCA Protein Assay Kit (Thermo Fisher Scientific) and then boiled for 10 min. Sample proteins were separated on 8%–12% sodium dodecyl sulfate-polyacrylamide gels and transferred to polyvinylidene difluoride membranes (Millipore, Darmstadt, Germany) by electrophoresis. The membranes were incubated overnight at 4°C with primary antibodies: anti-SESN2 (1:1000; Proteintech, Hubei, China), anti-Cyclin D, anti-β-actin, anti-fibronectin, anti-α-SMA (1:1000; Abcam, MA, USA), anti-E-cadherin, anti-LC3B, anti-N-cadherin, anti-p-ULK, anti-p-AMPK, anti-AMPK, anti-p-mTOR, anti-mTOR (1:1000; Cell Signaling Technology), anticyclin B1, and anti-p62, ATG7 (1:1000; Santa Cruz, NC, USA). Thereafter, the membranes were with horseradish peroxidase-conjugated IgG (anti-rabbit or anti-mouse) secondary antibody (1:5000, GenDEPOT, Katy, TX, USA) at RT for 2 h. The protein bands were visualized using a CS Analyzer 4 (ATTO, Tokyo, Japan).

### 2.5. Cell Proliferation Assays

Cell viability was evaluated using the EZ-Cytox cell viability assay kit (DoGen, Seoul, South Korea), according to the manufacturer's instructions. PC3 and DU145 cells were plated in 96-well plates with a 100-μL volume at a density of 1 × 10^4^ cells/mL. After 24, 48, and 72 h, 10 μL EZ-Cytox cell viability assay kit was added to each well. Absorbance was measured at 450 nm using a microplate reader (INNO, LTek, Gyeonggi-do, Korea).

### 2.6. Cell Immunofluorescence Staining

For immunofluorescence staining, PC3 and DU145 cells were seeded and fixed with 4% paraformaldehyde for 10 min. Then, the cells were blocked with 3% bovine serum albumin for 30 min, followed by incubation with the indicated primary antibodies, KI67 (1:200; Abcam) and LC3B (1:200; Cell Signaling Technology), for 1 h. Then, a secondary antibody labeled with fluorescein was added for 30 min. Finally, 4,6-diamidino-2-phenylindole (Vector Labs, CA, USA) was used to stain the nuclei. The images were acquired using a microscope (BX53; Olympus, Tokyo, Japan).

### 2.7. Wound Healing Assay

PC3 and DU145 cells were seeded in 12-well plates at a density of 5 × 10^5^ cells/well. The next day, a straight line was scratched onto the cell monolayer using a 200-μL pipette tip. After washing the cells with cold PBS, fresh culture medium containing 10% FBS was added. Images of migratory cells were captured at 0 and 24 h using an Olympus microscope. The relative cell migration rate of each group was normalized to the scratched areas at 0 h using ImageJ software (National Institutes of Health, MD, USA).

### 2.8. Transwell Assay

Cell migration and invasion capacity were evaluated using a 24-well cell culture plate with a Falcon 8-μm-pore-size chamber insert (Corning, NY, USA). Cells were seeded in serum-free RPMI-1640 in the upper chamber at a density of 5 × 10^4^ cells/well, and medium with 10% FBS added to the lower chamber. For invasion assays, Matrigel was diluted 1:10 with medium before seeding and was coated with 30 μL and allowed to coagulate for 2 h at 37°C. After 24 h, the cells in the upper chamber were removed using a cotton swab. The lower surface of the insert was fixed in 70% ethanol for 10 min and stained with 0.1% crystal violet staining solution containing methanol in PBS for 10 min. Migrated cells were counted under microscope (Olympus, Tokyo, Japan) at 200 × magnification.

### 2.9. Statistical Analyses

All statistical analyses were performed using Prism Version 5 (GraphPad Software, CA, USA). One-way analysis of variance or a *t*-test was used to evaluate significant differences between two groups of data. *p* ≤ 0.05 was considered statistically significant. All experiments were repeated at least three times.

## 3. Results

### 3.1. SESN2 Expression in Prostate Cancer Tissue and Cells

**Figure 1:**
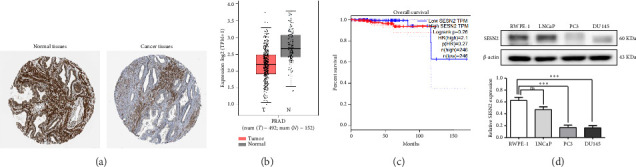
SESN2 expression level in prostate cancer tissues and cell lines. (a) SESN2 expression levels in prostate cancer and normal prostate tissues were analyzed from the human protein atlas databases. (b) SESN2 expression in prostate cancer patient tissues, as obtained from GEPIA databases. (c) Kaplan–Meier survival analysis showing the association between SESN2 expression levels and overall survival in 492 prostate cancer patients. (d) SESN2 expression levels in the normal prostate epithelial cell line RWPE-1 and prostate cancer cell lines (PC3 and DU145). T: tumor (red bar); N: normal (gray bar), ^∗^*p* < 0.05, ^∗∗^*p* < 0.01, and ^∗∗∗^*p* < 0.001.

We analyzed the expression of SESN2 in prostate cancer tissues and cell lines. In Figure 1(a), we identified reduced SESN2 expression in prostate cancer tissues compared to normal tissues using data from the Human Protein Atlas database. Similarly, in Figure 1(b), we observed decreased SESN2 mRNA levels in tumor tissues compared to normal tissues using the GEPIA database. Furthermore, a Kaplan–Meier survival analysis performed with the GEPIA database demonstrated that low SESN2 expression is associated with a reduced overall survival rate in prostate cancer patients (Figure 1(c)). Then, we confirmed SESN2 expression in prostate cancer cell lines. Western blot analysis revealed significantly lower SESN2 expression in prostate cancer cell lines (PC3 and DU145) compared to normal prostate cells (RWPE-1) (Figure 1(d)). Among the cancer cell lines, PC3 and DU145 exhibited particularly reduced SESN2 expression, which led us to select these cell lines for experiments. These findings indicate reduced SESN2 expression in human prostate cancer tissues and cell lines.

### 3.2. SESN2 Inhibits the Proliferation of Prostate Cancer Cells

**Figure 2:**
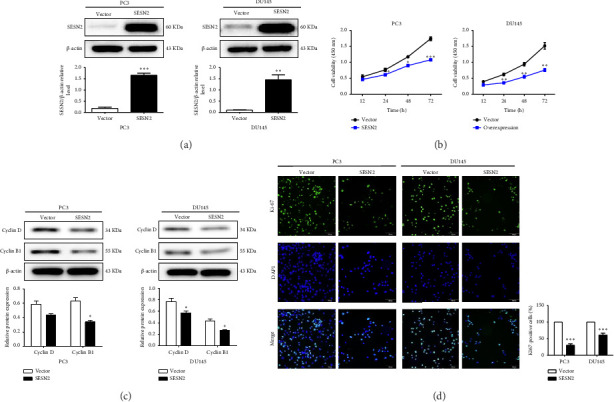
SESN2 inhibits prostate cancer cell proliferation. (a) PC3 and DU145 cells were transfected with SESN2 overexpression vectors and negative control (NC) vectors for 48 h. (b) Cell viability analysis with EZ-Cytox after transfection of PC3 and DU145 cells with control vectors and SESN2 overexpression vectors for 12, 24, 48, and 72 h. (c) Western blot analysis for proliferation-associated proteins. Protein expression was normalized using β-actin. (d) Immunofluorescence images and graphs displaying KI67 expression in PC3 and DU145 cells after transduction. Scale bars: 100 μm. ^∗^*p* < 0.05, ^∗∗^*p* < 0.01, and ^∗∗∗^*p* < 0.001.

The effects of SESN2 on prostate cancer cell proliferation were investigated. We show that SESN2 overexpressed in PC3 and DU145 cells and confirmed the protein expression levels of SESN2 (Figure 2(a)). Cell viability rates were determined using an MTT assay. Both PC3 and DU145 cells transfected with the SESN2 overexpression vector exhibited reduced growth rates compared with those transfected with the negative control vector (Figure 2(b)). This indicates that cell viability increased gradually over time but did not affect the differences observed between the groups. Then, we investigated protein markers associated with cell proliferation. We observed that the protein expression levels of Cyclin D and Cyclin B1 were significantly reduced in SESN2-overexpressed PC3 and DU145 cells compared to the control vector cells (Figure 2(c)). In addition, we analyzed the expression of the cell proliferation marker Ki67 in prostate cancer cells by immunofluorescence staining. In both cells, the expression of Ki67 was reduced in the SESN2-overexpressed cells (Figure 2(d)). The results showed that the overexpression of SESN2 inhibits the cell proliferation of PC3 and DU145 cells.

### 3.3. SESN2 Inhibits the Epithelial–Mesenchymal Transition (EMT) in Prostate Cancer Cells

**Figure 3:**
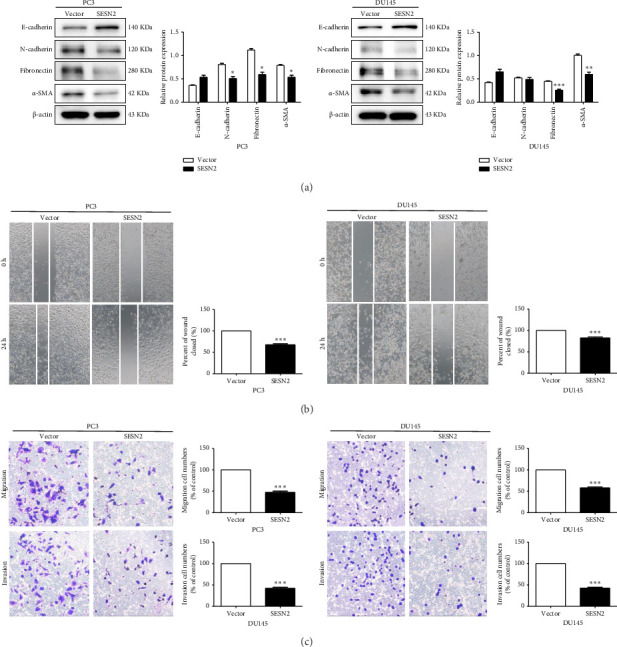
SESN2 decreases the migration and invasion of prostate cancer cells. (a) Western blot analysis of epithelial–mesenchymal transition (EMT)–associated protein levels. (b) Wound-healing assay to assess the mobility of PC3 and DU145 cells. (c) Transwell assay to evaluate cell invasion and migration. ^∗^*p* < 0.05, ^∗∗^*p* < 0.01, and ^∗∗∗^*p* < 0.001.

EMT is important for the initiation and development in cancer cell [14]. We observed EMT-related markers in prostate cancer cells. SESN2-overexpressed cells showed that the expression of mesenchymal-associated proteins marker (N-cadherin, fibronectin, and α-SMA) decreased and the protein levels of epithelial-associated proteins marker (E-cadherin) increased compared to control vectors cells (Figure 3(a)). We observed the effects of SESN2 on the migration and invasion of PC3 and DU145 cells using wound-healing and transwell assays. SESN2-overexpressed cells reduced wound closure and migration and invasion through the membrane in PC3 and DU145 cells compared to the control vector cells (Figures 3(b), 3(c)). These results indicate that overexpression of SESN2 may inhibit EMT, thereby reducing invasion, migration, and wound healing in prostate cancer cells.

### 3.4. SESN2 Enhances Autophagy in Prostate Cancer Cells

**Figure 4:**
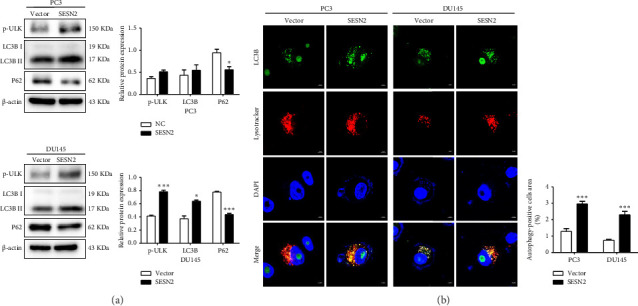
SESN2 induces autophagy in prostate cancer cells. (a) Western blot analysis of LC3B-I/II, p62, and p-ULK protein levels. (b) Immunofluorescence staining images and graphs of LysoTracker Red and LC3B staining in SESN2-overexpressed PC3 and DU145 cells, observed via fluorescence microscopy (200 × magnification). ^∗^*p* < 0.05, ^∗∗^*p* < 0.01, and ^∗∗∗^*p* < 0.001.

Autophagy can regulate tumor metastasis, which regulates tumor cell survival and proliferation [15]. Autophagy is a highly regulated cellular degradation process that plays a crucial role in maintaining cellular homeostasis by degrading and recycling damaged organelles and misfolded proteins. Proteins such as p62, LC3B, and ULK are well-known markers for identifying the process of autophagy. The conversion of LC3 from its cytosolic form (LC3-I) to its membrane-bound form (LC3-II) is a hallmark of autophagosome formation [16]. To investigate the role of SESN2 in autophagy, we examined the expression of key autophagy-associated proteins in PC3 and DU145 cells. In Western blot analysis, SESN2 overexpression led to an increase in the phosphorylation of ULK (p-ULK), indicating the activation of the upstream autophagy initiation complex (Figure 4(a)). In addition, SESN2 overexpression increased the conversion of LC3B-I to LC3B-II. This suggests that SESN2 promoted LC3B-I to LC3B-II, resulting in increased autophagosome formation. In contrast, p62 protein levels were significantly reduced, further confirming enhanced autophagic activity. To evaluate the formation of autophagosomes and lysosomal fusion, we performed immunofluorescence staining using LysoTracker (Thermo Fisher Scientific) and LC3B. Colocalization analysis demonstrated a significant increase in LysoTracker-positive areas (red) and LC3B puncta-positive regions (green) in SESN2-overexpressing cells compared to control vector cells (Figure 4(b)). This colocalization suggests that SESN2 overexpression promotes autophagosome–lysosome fusion, a key step in autophagic flux. Collectively, these findings indicate that SESN2 enhances autophagy in prostate cancer cells by modulating key autophagy regulators, including ULK, LC3B, and p62, ultimately promoting autophagic activity.

### 3.5. SESN2 Regulates Proliferation, EMT, and Autophagy via the AMPK/mTOR Signaling Pathway

**Figure 5:**
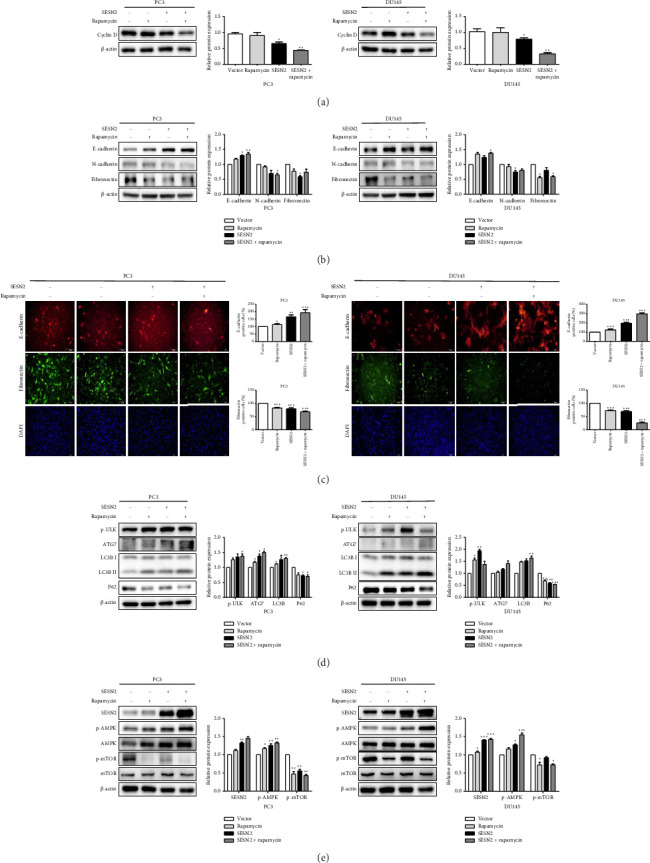
SESN2 regulates EMT and autophagy via the AMPK/mTOR signaling pathway in prostate cancer cells. (a) Western blot analysis of Cyclin D expression in prostate cancer cells. PC3 and DU145 cells were treated with rapamycin (10 nM) after transfection with an SESN2 overexpression vector. (b) Western blot analysis of E-cadherin, N-cadherin, and fibronectin in PC3 and DU145 cells. (c) Immunofluorescence staining images and graphs of E-cadherin and fibronectin expression in PC3 and DU145 cells. (d) Western blot analysis of LC3B-I/II, p62, ATG7, and p-ULK protein expression. (e) Western blot analysis of p-AMPK, AMPK, p-mTOR, and mTOR protein expression. ^∗^*p* < 0.05, ^∗∗^*p* < 0.01, and ^∗∗∗^*p* < 0.001.

SESN2 reduces mTOR phosphorylation [17]. We examined that proliferation, EMT, and autophagy were modulated by SESN2 via the AMPK/mTOR pathway following treatment with rapamycin, which is an mTOR inhibitor and activators for autophagy, in PC3 and DU145 cells. Rapamycin-treated SESN2-overexpressed cells was reduced significantly Cyclin D compared to SESN2-overexpressed cells (Figure 5(a)). Then, we investigated EMT-associated markers after rapamycin treatment via Western blot. After rapamycin-treated SESN2-overexpressed cells, the protein level of E-cadherin increased and N-cadherin and fibronectin decreased compared to SESN2-overexpressed cells without rapamycin treatment (Figure 5(b)). The effect of SESN2 on the EMT in prostate cancer cells was confirmed using immunofluorescence staining (Figure 5(c)). Rapamycin-treated SESN2-overexpressed cells showed an increase in the protein level of E-cadherin and decrease in the protein level of fibronectin compared with SESN2-overexpressed cells. Consistent with Figure 4(a), SESN2-overexpressed cells showed an increase in autophagy-related markers (p-ULK, ATG7, p62 and LC3B) in prostate cancer cells (Figure 5(d)). In rapamycin-treated SESN2-overexpressed cells, the protein level of p-ULK, ATG7, and LC3B were increased and that of p62 were significantly decreased compared to SESN2-overexpressed cells. To investigate the signaling pathway for SESN2 on autophagy activation, the expressions of AMPK and mTOR were analyzed (Figure 5(e)). Our results showed that SESN2-overexpressed cells increased protein level of p-AMPK and decreased ρ-mTOR in PC3 and DU145 cells. Furthermore, we showed that rapamycin-treated SESN2-overexpressed cells further increased the protein level of p-AMPK and further decreased mTOR in prostate cancer cells. These results suggest that SESN2 regulates cell proliferation, EMT, and autophagy by activating the AMPK/mTOR pathway.

## 4. Discussion

Prostate cancer is a threat to male health worldwide and has a poor prognosis [18]. Identifying potential genes that influence the development and progression of prostate cancer is crucial for advancing its diagnosis and treatment. In this study, we investigated the role of SESN2 in prostate cancer cells and the associated signaling pathways involved.

Cancer is widely recognized as an uncontrolled disease of cell proliferation and survival [19]. Recent studies have demonstrated that SESN2 plays a role in the development and progression of various human cancers. For instance, in colorectal, lung, and hepatocellular cancers, SESN2 expression is associated with proliferation, metastasis, and survival [20–22]. Specifically, Chet et al. reported that SESN2 expression was lower in tissue samples from patients with non–small-cell lung cancer compared to noncancerous lung tissues. This decreased expression correlated with lower differentiation, lymph node metastasis, and shorter overall survival [21]. Similarly, Wei et al. found that SESN2 was downregulated in colorectal cancer and that viral vector-mediated upregulation of SESN2 reduced proliferation, EMT, and colony formation in colon cancer cells [20]. However, little is known about the role of SESN2 in prostate cancer.

Consistent with reports from colorectal and lung cancers, our results revealed that SESN2 expression was significantly reduced in prostate cancer tissues and cell lines. Furthermore, low SESN2 expression was associated with a poor prognosis in prostate cancer patients. Our study confirmed that SESN2 expression is diminished in prostate cancer cell lines compared to normal prostate cells. Through SESN2 overexpression experiments, we demonstrated that SESN2 inhibits cell proliferation in prostate cancer cells.

EMT is a biological process where polarized epithelial cells lose their adhesion properties and acquire mesenchymal phenotypes. This transition is marked by an increase in mesenchymal markers, such as snail, N-cadherin, vimentin, fibronectin, and α-SMA, and a decrease in epithelial markers, such as E-cadherin and occluding [23]. EMT is critical in regulating cancer cell invasion, migration, and progression [24]. Previous studies have shown that SESN2 inhibits EMT in colorectal cancer [20]. In our study, SESN2 inhibited invasion and migration in prostate cancer cells, suggesting that it suppresses prostate cancer progression. Collectively, our findings suggest that SESN2 modulates EMT in prostate cancer cells.

Recent studies have highlighted a complex relationship between autophagy and EMT in cancer [14]. Autophagy, a self-degradation process, has shown to play a vital role in cancer cell metastasis. Autophagy involves the lysosome-dependent degradation of misfolded proteins and damaged organelles via double-membrane autophagosomes, which then fuse with lysosomes for recycling [15]. The AMPK/mTOR signaling pathway is a key regulator of autophagy in cancer cells [25, 26]. Elevated phosphorylated AMPK levels and decreased phosphorylated mTOR levels induce autophagy in prostate cancer cells [27]. Autophagy-associated proteins, such as ULK1, ATG13, AMBRA1, and ATG14L, also promote the initiation and progression of autophagy [16, 26]. Importantly, autophagy is closely linked to SESN2 expression. Upregulation of SESN2 contributes to autophagy induction via activation of the JNK pathway, and SESN2 promotes autophagy in hippocampal neurons during sepsis-related encephalopathy [28, 29]. In our study, SESN2 overexpression increased autophagy-associated proteins such as p-ULK and LC3B, further enhancing autophagy activity. These findings suggest that SESN2 promotes autophagy activity in prostate cancer.

Activation of autophagy correlated with various signaling pathways, including increased AMPK phosphorylation and decreased mTOR phosphorylation. Moreover, activation of the AMPK/mTOR signaling pathway with rapamycin inhibited EMT and proliferation by increasing epithelial marker expression (e.g., E-cadherin) and reducing mesenchymal marker expression in prostate cancer cells. These findings indicate that SESN2 regulates autophagy in prostate cancer cells. Our data demonstrate that SESN2 activates autophagy and inhibits EMT and proliferation via the AMPK/mTOR pathway.

In conclusion, our study highlights that SESN2 induces autophagy and reduces cell proliferation by inhibiting EMT in prostate cancer cells. While further research is necessary, our findings suggest that SESN2 has potential anticancer effects in prostate cancer by modulating AMPK/mTOR-mediated autophagy.

Finally, the limitations of this study primarily focus on the role of SESN2 in prostate cancer cell lines, with a lack of in vivo validation in animal models. We have used various culture media to meet the specific growth requirements of cell lines, and further studies are needed to investigate the function of SESN2 in various subtypes and stages of prostate cancer. Additionally, the mechanisms underlying SESN2-induced autophagy remain incompletely understood. Further studies are required to assess the clinical applicability and safety of SESN2 modulation.

## 5. Conclusions

In summary, our results suggest that SESN2 modulates proliferation and EMT in prostate cancer cells, which may be related to the activation of AMPK/mTOR signaling pathway-mediated autophagy. This suggests that SESN2 has antitumor effects on human prostate cancer cells. As SESN2 is an important component in cancer, it may be worth further investigation for the treatment of prostate disease.

## Data Availability

The data that support the findings of this study are available from the corresponding author upon reasonable request.
